# Radiotherapy-related changes in serum proteome patterns of head and neck cancer patients; the effect of low and medium doses of radiation delivered to large volumes of normal tissue

**DOI:** 10.1186/1479-5876-11-299

**Published:** 2013-12-05

**Authors:** Piotr Widłak, Monika Pietrowska, Joanna Polańska, Tomasz Rutkowski, Karol Jelonek, Magdalena Kalinowska-Herok, Agnieszka Gdowicz-Kłosok, Andrzej Wygoda, Rafał Tarnawski, Krzysztof Składowski

**Affiliations:** 1 Maria Skłodowska-Curie Memorial Cancer Center and Institute of Oncology, Gliwice Branch, Gliwice, Poland; 2 Institute of Automatics Control, Silesian University of Technology, Gliwice, Poland; 3 Polish-Japanese Institute of Information Technologies, Warszawa, Poland

**Keywords:** Dose effects, Head and neck cancer, IMRT, Radiation toxicity, Serum proteome

## Abstract

**Background:**

Conformal intensity-modulated radiation therapy (IMRT) involves irradiation of large volume of normal tissue with low and medium doses, biological relevance of which is not clear yet. Serum proteome features were used here to study the dose-volume effects in patients irradiated with IMRT due to head and neck cancer.

**Methods:**

Blood samples were collected before and during RT, and also about one month and one year after the end of RT in a group of 72 patients who received definitive treatment. Serum proteome profiles were analyzed using MALDI-ToF mass spectrometry in 800–14,000 Da range.

**Results:**

Major changes in serum proteome profiles were observed between pre-treatment samples and samples collected one month after RT. Radiation-related changes in serum proteome features were affected by low-to-medium doses delivered to a large fraction of body mass. Proteome changes were associated with intensity of acute radiation toxicity, indicating collectively that RT-related features of serum proteome reflected general response of patient’s organism to irradiation. However, short-term dose-related changes in serum proteome features were not associated significantly with the long-term efficacy of the treatment.

**Conclusions:**

The effects of low and medium doses of radiation have been documented at the level of serum proteome, which is a reflection of the patient’s whole body response.

## Background

Radiotherapy (RT), either alone or in combination with chemotherapy, is an effective treatment of different types of cancer, including head and neck squamous cell carcinoma, which allows preserving the structure and function of a target organ. Successful RT of patients with advanced cancer usually requires aggressive methods of treatment, which include accelerated fractionation successfully implemented in radical treatment of head and neck cancer [1,2]. However, application of such a treatment increases risk of unacceptable toxicity to normal tissue, where radiation induces damage leading to acute and/or late injury reactions [3].

Differences in repair of radiation damage between tumor and normal tissue are the main biological concept of radiotherapy, and until the end of 20th century radiation therapy was applied using two opposite radiation fields with similar doses delivered to tumor and normal tissue (dose fractionation was used to reduce toxicity of such a treatment). In recent two decades development of advanced 3D radiation treatment planning systems and technological progress in construction of linear accelerators with multileaf collimators (MLC) allowed on real differentiation of radiation doses between tumor and normal tissue. The main concept of conformal radiotherapy is based on the focusing of several radiation fields in the target volume, allowing a higher dose of radiation to be delivered to the tumor and (intentionally) reducing its toxicity to the surrounding normal tissues. The intensity-modulated radiation therapy (IMRT) is the next step in optimization of radiation dose. This technique takes advantage of treatment planning systems by calculating the optimal position of MLC during irradiation, which results in better differentiation of dose between normal tissue and tumor [4]. Introduction of many non-coplanar radiation fields results in irradiation of larger volumes of normal tissues with small doses of radiation comparing to two-field technique, yet the increase in volume of patient’s body exposed to low doses of radiation is a potential drawback of IMRT. Theoretically irradiation with small doses should not introduce substantial damage to normal tissues because of a threshold in the dose-effect relationship. On the other hand, however, the whole body effect of irradiation using IMRT has not been studied in depth because of the lack of existing end-points and biological markers of irradiation to small doses. In fact, the only concern regarding IMRT that has been examined so far is a treatment of children in the context of a risk of secondary cancers. Hence, documenting the possible biological effects of irradiation of large masses of normal tissues with low and medium doses is an urgent issue rev. in: [5,6].

The low-molecular-weight fraction of serum/plasma proteome (up to 15,000 Da) appears to be a promising source of novel biomarkers rev. in: [7,8]. Several published studies on mass spectrometry-based profiling of this fraction of blood have revealed multi-peptide signatures that could be used for detection and/or classification of cancer. Noteworthy, several components of proposed cancer signatures, especially those characteristic for advanced cancer, were identified as fragments of blood proteins involved in general response of patient’s organism to the disease. Of note, profiling of serum/plasma proteome revealed features that reflected general reaction of the patient’s whole body to anticancer treatment [8-10]. A few published works used this approach aiming to detect radiation-related effects in locally irradiated cancer patients. These works reported changes in serum proteome profiles between pre-exposure and post-exposure samples, and found differences between samples from patients exposed to different doses of radiation [11,12]. Although both reports documented the influence of radiation doses on therapy-related changes in the serum proteome, they focused on the radiation doses delivered to tumor volume. Here, we hypothesized that the whole body response of a patient to the treatment may be related not only to high doses delivered to tumor volume but also to low- and medium-doses delivered during IMRT to large volume of normal tissue.

## Methods

### Characteristics of patient group

Seventy two patients with head and neck squamous cell cancer were enrolled into this prospective study; all participants were Caucasians (51 men), 40–80 years old (median age 60 years), mostly current smokers (85%) and alcohol consumers (83%). Cancer was located at either oropharynx (31), hypopharynx (8) or larynx (33); primary tumor stage was scored as: T1 (7%), T2 (43%), T3 (34%) and T4 (16%); 51% of N0. All patients were subjected to IMRT using 6 MeV photons (1.8 Gy daily fraction doses according to the continuous accelerated irradiation scheme, CAIR [2]). Total radiation doses delivered to gross tumor volume (GTV) were in the 66.6-73.8 Gy range (median 72 Gy), overall treatment time was between 38–49 days (median 41 days). Neither surgery nor induction/concomitant chemotherapy was applied to patients enrolled in the study. The acute mucosal reaction (AMR) was assessed using the Dische score system [13,14] each 3–5 days during the RT. Three-year recurrence-free survival was observed in 36 patients, while treatment failure due to local recurrence, distant metastases and/or second tumor was noted in 25 patients during the same follow-up. Four consecutive blood samples (5 ml) were collected from each patient: pre-treatment sample A (within one week before RT; 72 patients); within-treatment sample B (9–23 days after the start of RT; median 15 days; 72 patients); post-treatment sample C (16–73 days after the end of RT; median 34 days; 68 patients) and post-treatment sample D (52–714 days after the end of RT; median 460 days, 59 patients). The study was approved by local Ethics Committee at the Cancer Center in Gliwice and all participants provided informed consent indicating their conscious and voluntary participation.

Irradiated volumes and related radiation doses were read from treatment plans that based on CT scans of head, neck and upper thorax region (using the ECLIPSE radiation therapy planning system). Because the CT scans always include region containing all radiation fields assessment of doses and volumes is straightforward. The dose-volume histograms (DVH, see below) represent volume of irradiated tissue irradiated with specific dose level (according to the treatment plans), which is an important element of evaluation of IMRT plan.

### Sample preparation and serum mass profile acquisition

Collected blood was incubated for 30 min. at room temperature to allow clotting, and then centrifuged at 1000 g for 10 min. to remove the clot. The serum was aliquoted and stored at -70°C. Directly before analysis samples were diluted 1:5 with mixture of acetonitrile (20%) and ammonium bicarbonate (250 mM), and then albumin and other large-molecular-weight proteins were removed by centrifugation (30 minutes, 3000 g) through AmiconUltra filters with 50 kDa cut-off membrane (Millipore). Each sample was loaded onto a C18 ZipTip micro-column by passing it through repeatedly 15 times until saturation, then column was washed with water and eluted with 1 μl of matrix solution (saturated solution of alpha-cyano-4-hydroxycinnamic acid in 50% ACN/H_2_O and 0.1% TFA) directly onto the 600 μm AnchorChip (Bruker Daltonics) plate. Samples were analyzed using an Ultraflextreme MALDI-ToF spectrometer (Bruker Daltonics, Bremen, Germany); the analyzer worked in the linear mode and positive ions were recorded in the mass range between 800 and 14,000 Da. ZipTip extraction/loading was repeated twice for each sample and for each spot two averaged spectra were recorded (i.e. four spectra were acquired for each sample). All samples were analyzed in a random sequence to avoid a possible batch effect.

### Analysis of mass profiles

The pre-processing of spectra, which included baseline removal, alignment and averaging of technical repeats, and normalization of the total ion current (TIC), was performed according to procedures considering to be standard in the field [15]. In the second step the spectral components, which reflected [M+H]^+^ peptide ions recorded at defined m/z values, were identified using decomposition of mass spectra into their Gaussian components followed by several post-processing steps as described in more details elsewhere [16,17]. The average spectrum was decomposed into a sum of Gaussian bell-shaped curves by using a variant of the expectation maximization (EM) algorithm and Bayesian Information Criterion (BIC) for model selection [18]. The initial set of 366 Gaussian components, defined by their mean values and standard deviations, was further processed to merge overlapping components (components homogenous in variance and with main values closer than 0.1% of the m/z value) and to remove components presumably representing the residual baseline (components with coefficient of variation bigger than 25%), which resulted in dimension reduction to 319 Gaussian components. The final 319 Gaussian components were used to compute features of registered spectra (termed spectral components afterward) for all samples by the operations of convolutions with Gaussian masks. The knowledge base EPO-KB (Empirical Proteomic Ontology Knowledge Base) [19], which links m/z values to known peptide/proteins, was used for hypothetical annotation of spectral components.

### Statistical analyses

For each serum component the normality of distribution of changes was assessed using the Lilliefors test to provide optimal tools for statistical analysis. Either the t-test or the Wilcoxon test corrected for multiple testing with false discovery rate (FDR) estimation was applied for analyzing changes in abundances of spectral components. Correlation between abundance of spectral components and parameters reflecting absorbed doses of radiation was analyzed using either the Pearson’s correlation coefficient or the Spearman’s rank correlation coefficient (depending on the type of distribution). Total radiation energy absorbed by patient’s body was estimated from the individual DVH generated during the treatment planning by calculating its integral over dose. For the analysis of dose/volume effect doses corresponding to deciles of the area under curve of the averaged DVH were selected (see below) Correlation between maximal intensity of the AMR and abundance of spectral components or radiation doses was analyzed using the Spearman’s rank correlation coefficient.

## Results

### Exposure to ionizing radiation resulted in gross changes of the low-molecular-weight component of serum proteome

**Figure 1:**
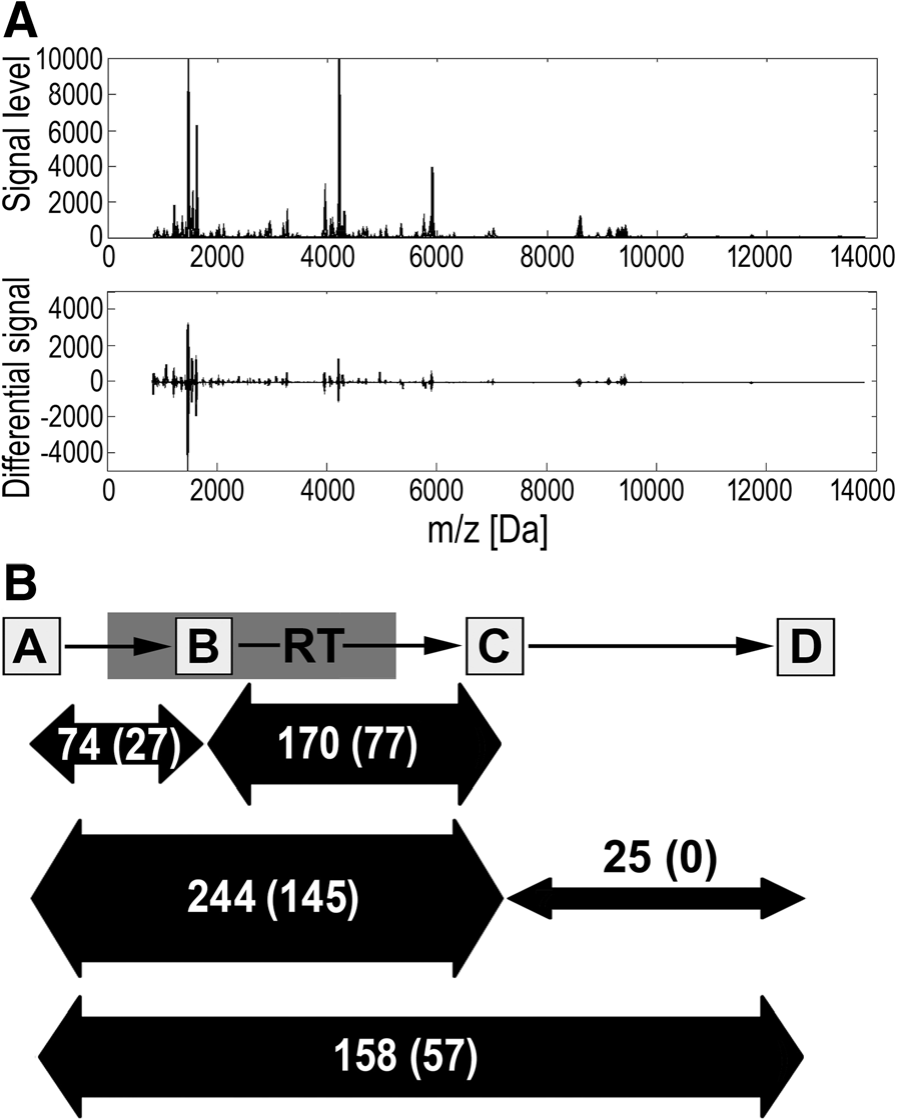
**Radiation-induced changes in serum proteome profiles.** Panel **A** - Typical mass spectrum of the serum proteome in the 800–14,000 Da range and differential spectrum computed for samples collected before and during radiotherapy. Panel **B** - Numbers of spectral components that changed their abundances between consecutive samples at high level of statistical significance (FDR < 5% or FDR < 0.1%, the latter ones in brackets).

A typical MALDI-ToF mass spectrum registered in the 800–14,000 Da range (319 spectral components or peptide ions were distinguished in this range) and a differential spectrum obtained for two consecutive samples is exemplified on Figure 1A. Pairwise differential spectra were computed for consecutive samples collected from each donor, and then the statistical significance of differences in peptide abundances was estimated. Numbers of spectral components that changed their abundances between compared time points are presented in Figure 1B (detailed data are presented in Additional file 1: Table S1). The B samples represented the first weeks of radiotherapy after accumulation of about 1/3 of total dose when the first signs of radiation toxicity were visible. The C samples reflected early effects of the completed treatment when total radiation dose was delivered and healing of radiation mucositis was observed. The D samples reflected longer follow-up where early radiation effects were completely healed and late effects of RT on both normal mucosa and tumor (cancer eradication or ongoing recurrence) might be detected. Several serum components (~8% of detected peptide ions) changed their abundance at a high level of statistical significance (FDR < 0.1%) during the first two weeks of radiotherapy (the AΔB change). However, the major changes were observed when post-treatment serum samples C were analyzed. About 45% of detected peptide ions changed their abundance significantly (FDR < 0.1%) between samples A and C, while about 24% of peptide ions changed their abundance between samples B and C. A substantial part of changes observed during the first two weeks of RT deepen until sample C, however, majority of changes (~70%) observed one month after the end of RT (sample C) were not detected earlier after the first two weeks of RT. Changes observed during a longer follow-up (the CΔD change) were apparently less prominent. We concluded that exposure to ionizing radiation resulted in gross changes detected primarily early after the end of the treatment. Importantly, the treatment-related changes could be detected several months after the end of treatment, hence abundances of about 18% of detected peptide ions were still significantly different (FDR < 0.1%) when samples collected before the treatment (sample A) and more than one year after the end of RT (sample D) were compared. Of note, we did not observe a statistically significant association of RT-induced changes of serum proteome features with cancer stage or location.

### Radiation-induced changes in the serum proteome profiles could be reversed during long time follow-up

**Figure 2:**
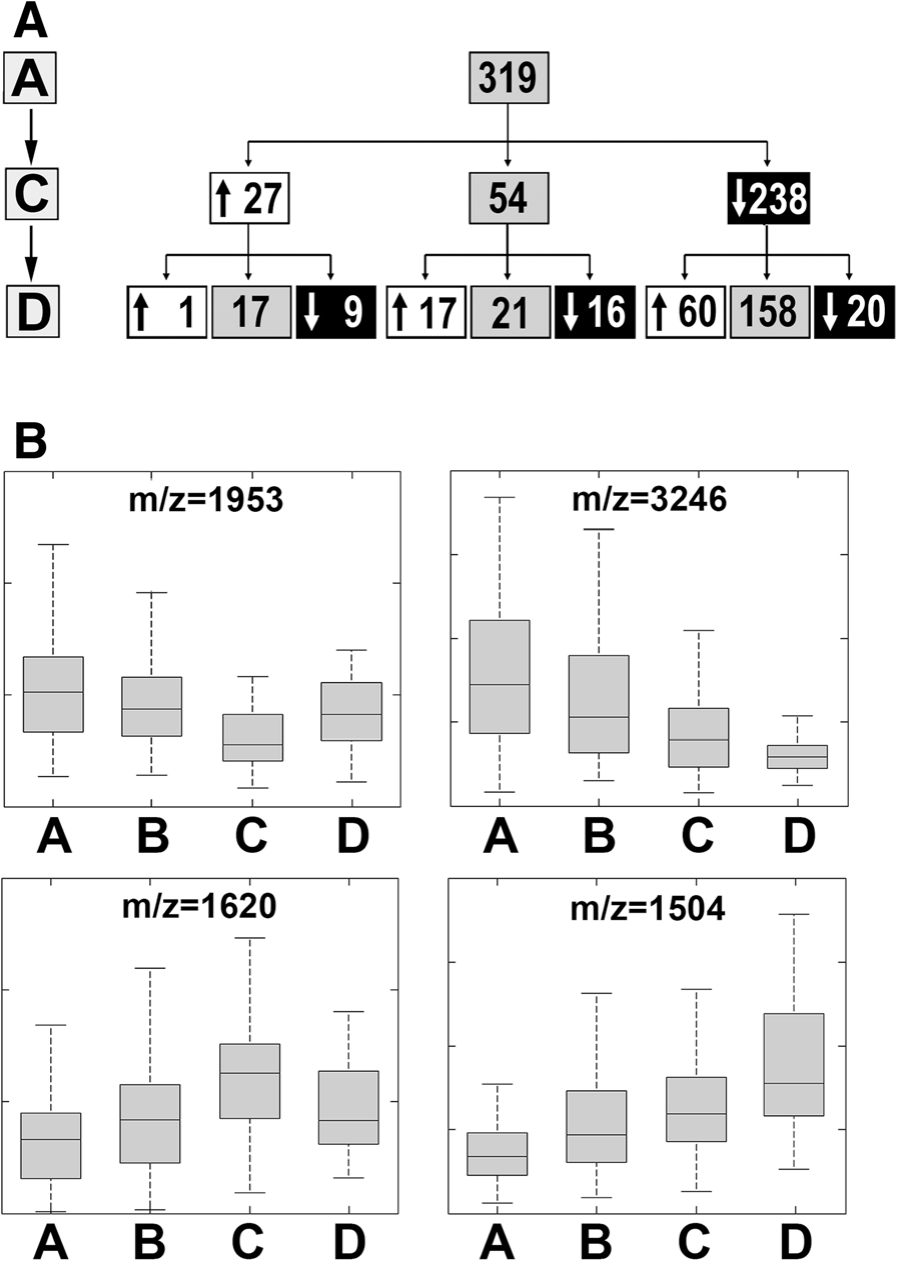
**Radiotherapy-related changes in patterns of serum proteome features.** Panel **A –** The numbers of spectral components that followed particular patterns of changes between analyzed time points (either decrease and increase marked with corresponding arrows, or lack of statistically significant differences). Panel **B** – Examples of registered peptide ions that represented four specific patterns of changes; boxplots show: minimum, lower quartile, median, upper quartile and maximum values.

We analyzed kinetics of radiotherapy-related changes in serum proteome patterns during the longer follow-up. Diagram in Figure 2A shows the numbers of peptide ions that changed their abundance following specific patterns; “early” changes detected between samples A and C, and “late” changes detected between samples C and D were analyzed (p = 0.05 was selected as a significance threshold for increased or decreased abundance). We observed that substantially more serum components decreased in their abundances between samples A and C (238 out of 319) than increased in their abundances between these samples (27 out of 319). Interestingly, “early” changes detected during short time after the treatment (the AΔC change) appeared to be somehow reversed/compensated by “late” changes observed during longer follow-up (the CΔD change). Such “reversing” effect was much more frequent than “deepening” of changes: 60 vs. 20 and 9 vs. 1 components that either decreased or increased their abundance during the “early” phase, respectively; only in case of a few serum components changes induced during the treatment (or short time after the end of RT) were deepen during longer follow-up. Figure 2B shows examples of peptide ions representing different patterns of changes observed during the treatment and its follow-up (with “late” changes that either reverse or deepen “early” changes); namely abundances of components with m/z 1504 Da, 1620 Da, 1953 Da and 3246 Da are shown. Observed changes in serum proteome profiles suggest that RT-induced effects are related mostly to accumulation and healing of early radiation effects (e.g. radiation mucositis).

### Treatment-related changes in serum proteome profiles were associated with volumes of irradiated tissues and doses of radiation received by patients

**Figure 3:**
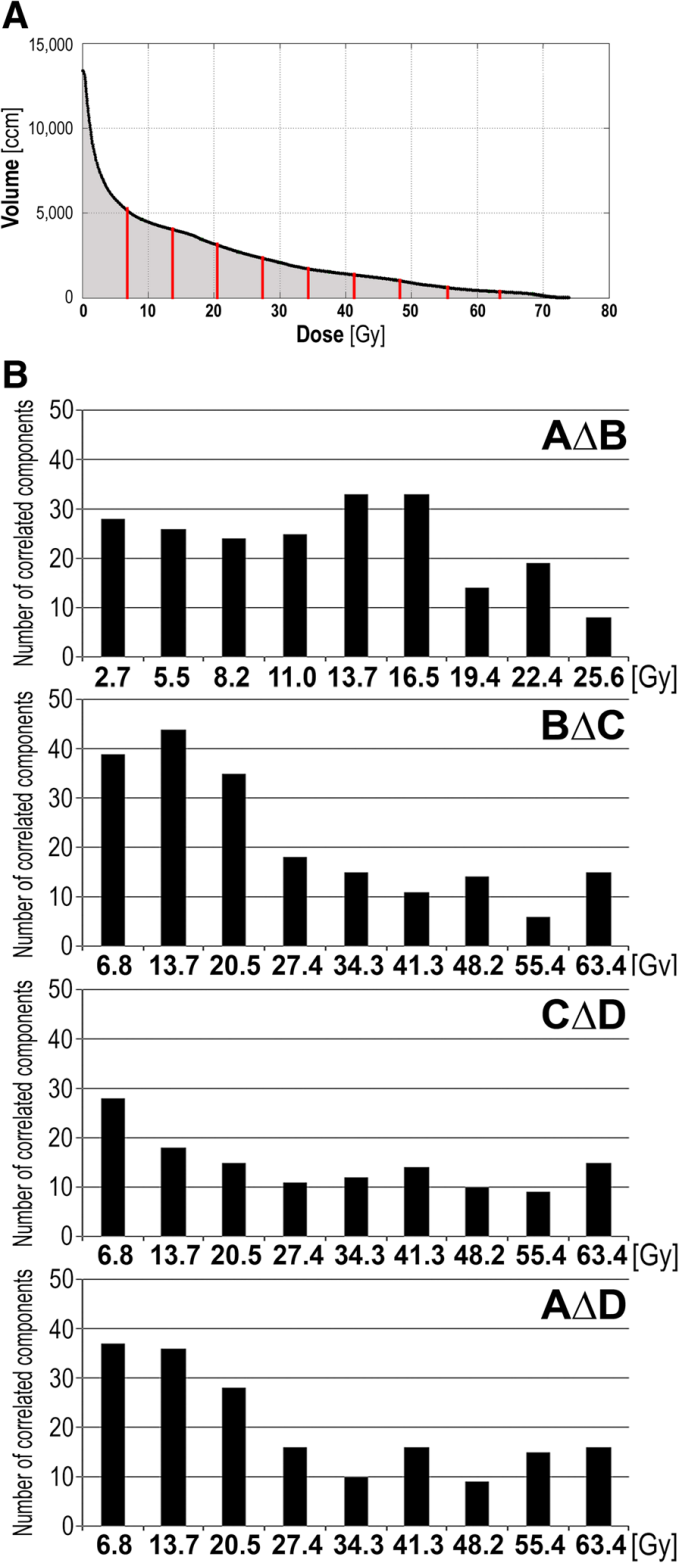
**Dose-volume effects in serum proteome changes. **Panel **A** – Averaged Dose-Volume Histogram; marked are doses corresponding to deciles of the area under curve of the histogram. Panel **B** - Numbers of serum proteome features correlating with tissue volume irradiated at a given dose for the AΔB, BΔC, CΔD and AΔD changes; p = 0.05 was selected as a statistical significance threshold.

In the second step of the study we searched for association between features of serum proteome profiles and doses of ionizing radiation received by patients (doses accumulated until a time point corresponding to the collection of sample B in case of the AΔB changes and total doses in case of the AΔC, BΔC and CΔD changes). Correlations were identified between specific changes in abundances of the detected peptide ions (i.e., features of serum proteome) and either the total (maximum) dose received by the gross tumor volume (GTV), volume of the patient’s body irradiated at different (smaller) doses or total radiation energy absorbed by patient’s body. The total doses are regarded as maximum tolerated doses, and are delivered to tumor and its adjacent margins (usually 100–200 ccm). Total radiation energy absorbed by patient’s body was estimated from the individual DVH by calculating its integral over dose (radiation dose multiplied by mass approximated from volume of irradiated tissue reflected absorbed energy); such energy was usually up to 300 J. Table 1 shows the numbers of serum components, which changes in abundances correlated with either maximum GTV dose or total absorbed radiation energy; p = 0.05 was selected as a statistical significance threshold. We found that changes in abundances of several serum components correlated with both maximum doses delivered to the target tumor volume and total radiation energy absorbed by whole patient’s body, and could be detected for both “early” changes (during and soon after the treatment) as well as “late” changes (during long-term follow-up). In general, more serum features correlated with the total radiation energy absorbed by the whole patient’s body (that in large part reflected doses delivered to normal tissue) than with the maximum GTV dose. Hence, effects related to a high dose of radiation received by a small volume of tumor and adjacent tissue (about 70 Gy in this case), and clinically/therapeutically low-to-medium doses received by a large volume of normal tissue were compared. Due to the characteristics of IMRT, tissue irradiated with lower doses represent much higher volumes (e.g. about 4,000 ccm irradiated with 10 Gy; see Figure 3A). Detailed data on correlation of serum proteome features with volumes irradiated at a given dose or with doses delivered to a given volume are presented in Additional file 1: Tables S2-S6. We found that “low- and medium-dose effects” were more frequent than “high-dose effects” when RT-induced serum proteome changes were analyzed (Figure 3B). Medium-dose effects were observed when the first phase of the treatment was analyzed (the AΔB changes); the highest number of serum features correlated with the volume of tissues irradiated at about 1 Gy dose fractions. However, low-dose effects predominated when the second phase of the treatment was analyzed (the BΔC changes); the highest number of serum features correlated with the volume of tissues irradiated at 0.16-0.33 Gy dose fractions (cumulated dose 6.8 or 13.7 Gy). Similar effects of low doses were observed when the “late” CΔD changes or the “overall” AΔD changes were analyzed. In all cases a relatively low number of serum features correlated with the volume of tissues irradiated at high doses (i.e. 1.5-1.8 Gy dose fractions). The same results were obtained when reverse analysis was performed - serum proteome features were correlated with a dose delivered to a given volume (Additional file 1: Tables S2-S6). All these observations indicated coherently that changes observed in the serum proteome patterns during and early after RT were primarily affected by low-to-medium doses of radiation absorbed by large fractions of patient’s body mass.

**Table 1:** Numbers of serum components correlated with GTV doses, total absorbed radiation energy (TARE) and maximum AMR intensity

**Change**	**Number of correlated components**			**Examples of components (m/z and hypothetical annotation)**
	**GTV-D**	**TARE**	**AMR**	
AΔB	4	26	19	4894, 6282 [CYTC], 9715
BΔC	13	29	11	1192, 1209, 1266 [A4]
AΔC	4	5	11	5194, 5215
CΔD	13	17	10	2110 [A4], 2994
AΔD	4	32	10	1334 [FIBA], 1433, 5407 [S10A8]

Presented are examples of components showing significant correlation with both AMR and radiation doses; p = 0.05 was selected as a significance threshold level.

### Changes in serum proteome profiles were associated with toxicity of the treatment

**Figure 4:**
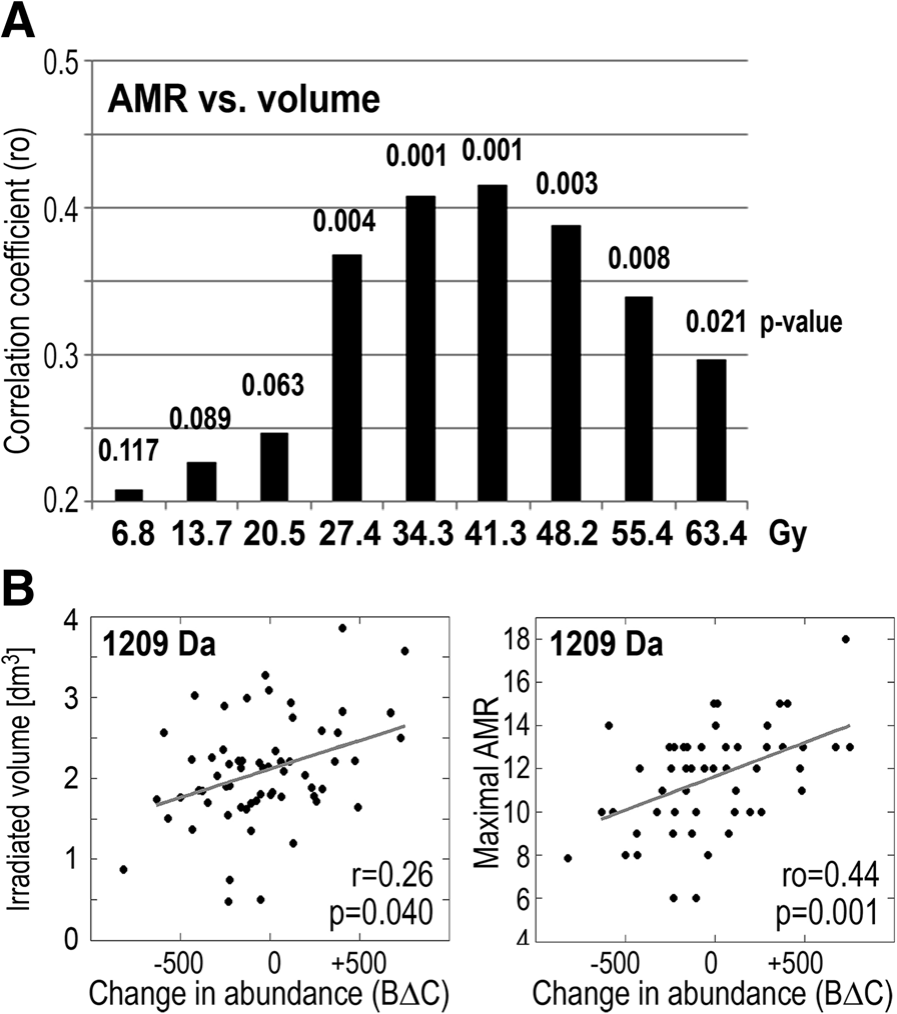
**Dose-effect in radiation response.** Panel **A** – Correlation between the maximum AMR intensity and volume of tissue irradiated a given dose; bars represent correlation coefficients while their p-values are shown on top. Panel **B** – Correlations between changes in abundance of the serum component m/z = 1209 Da in samples collected during and after RT (C-B; arbitrary units) and volume irradiated with 34.3 Gy or the maximum AMR intensity.

Aiming to examine presumable association of serum proteome with radiation toxicity we searched for correlation between dose-related changes in serum proteome features and clinical response to the treatment. The intensity of the acute mucosal reaction (AMR), which is a clinically relevant response of normal tissues to early toxicity of radiation [3] was assessed using a modified Dische system [13,14]. First, we have established correlation between the maximum AMR intensity and absorbed radiation doses. The maximum AMR intensity varied individually and was usually observed 20-40 days after the start of RT, yet linear increase of all irradiation parameters justified establishing correlation between the AMR and the total dose. As expected, we found positive correlation between the maximum AMR intensity and the GTV dose (ro = 0.30, p = 0.025) or total radiation energy absorbed by patient’s body (ro = 0.35, p = 0.008). When we looked for different dose effects the strongest association of the maximum AMR intensity was observed with “intermediate” doses of radiation (corresponding to 0.8-1 Gy dose fraction) delivered to substantial volumes of normal tissues (1-2 dm^3^); Figure 4A. Knowing the correlation between the AMR and delivered radiation doses, we searched for its association with changes of serum proteome features. We found that changes in abundances of several serum components correlated with the maximum AMR intensity; such correlation was the most frequent for the AΔB changes (Table 1). Furthermore, a substantial fraction of serum proteome components, which changes correlated with the maximum AMR intensity, also showed significant association with absorbed radiation doses. This phenomenon could be exemplified by the peptide ion m/z = 1209 Da, which is showed in Figure 4B. The data indicated collectively that radiotherapy-related changes in the serum proteome were affected by radiation doses delivered to large volumes of normal tissues and reflected biological mechanisms induced upon irradiation (e.g. acute radiation toxicity). We also searched for potential association between RT-induced changes of serum proteome features and the overall efficacy of the treatment. Two sub-groups were compared: patients with three-year recurrence-free survival and patient with local or distant failure diagnosed during the same time. Table 2 shows the numbers of serum components, which RT-induced changes in abundance were significantly different between these two sub-groups. Changes of serum proteome features observed long time after the treatment (i.e., the CΔD change) contributed primarily to such differences. This effect resulted mainly from significant difference in levels of analyzed components in sample D; there were 48 components in sample D with significantly different levels between compared subgroups. Of note, none of the serum proteome components that differentiated analyzed sub-groups of patients correlated with either received doses of radiation or maximal intensity of acute radiation toxicity (i.e., the maximal AMR). Hence, these features of serum proteome profiles observed after long time follow-up putatively reflected the tumor remission or relapse, but not direct effects of irradiation.

**Table 2:** Numbers of spectral components associated with efficacy of the treatment

**Change**	**Number of components**	**Examples of components (m/z and hypothetical annotation)**
AΔB	4	-
BΔC	10	5737, 5755 [SAA1], 5765
AΔC	5	-
CΔD	38	1953, 2192 [HEPC], 2354, 2647, 3579
AΔD	4	-

Numbers of components, for which changes in abundances were different between subgroups of patients with successful treatment or treatment failure; p = 0.05 was selected as a significance threshold level. Presented are the examples of components, for which significance of differences between sub-groups was p < 0.005.

## Discussion

We showed here that local irradiation of patients during the course of head and neck cancer radiotherapy resulted in gross changes in the low-molecular-weight component of the serum proteome after the end of RT. The relatively smaller changes in the serum proteome that were detected during the first phase of RT could be explained by a lag period and/or certain dose threshold-requirements of radiation-induced processes. This observation was coherent with the results of a study performed on a smaller group of patients treated due to larynx cancer [12]. In the present study we focused on dose effects and aimed to assess the importance of clinically low and intermediate doses of radiation delivered to large fractions of normal tissues. Two previous studies concerning the impact of local cancer irradiation on the serum proteome, which revealed the importance of radiation doses, focused on maximum doses delivered to tumor volume and based their conclusions on rather heterogenous clinical material [11,12]. Here, we analyzed homogenous material obtained from a relatively large group of patients where no cancer surgery or chemotherapy was applied. Hence, the heterogeneous factors that could interfere with the obtained results were excluded. We observed that large overall extent of massive changes induced upon the irradiation apparently impaired ability to detect more refined quantitative correlations with radiation doses. Nevertheless, association of specific changes in abundance of serum components with doses and volumes of irradiated tissue was found. Although correlations identified between specific proteome features and different parameters reflecting radiation doses possess moderate statistical power when analyzed separately, reliable conclusions could be drawn based on the general patterns of observed association. We noted that effects of low-to-medium doses delivered to large volumes of normal tissue were apparently more frequent than effects of high doses delivered to target volume of tumor, and concluded that effects of such low-to-medium doses could be observed at the level of whole body response to radiotherapy.

We found that massive changes of serum proteome profiles observed during and soon after the end of radiotherapy, i.e. “early” effects of irradiation, were somehow reversed/compensated by “late” changes, observed at longer times of the follow-up. This apparently reflected process of return to the initial steady-state level (e.g. accumulation and subsequent healing of radiation damage). However, “late” radiation-induced changes observed during the follow-up were relatively weaker and not always fully reversed the stronger changes induced during the treatment. As a consequence, radiation-related changes could be observed months after the end of radiotherapy and abundances of several serum components were significantly different when samples collected more than one year after the treatment, were compared to samples collected before the radiotherapy. Similar observation was made in a group of breast cancer patients where changes in serum proteome profiles related to the adjuvant radio/chemotherapy were detected one year after the treatment [20]. Such results clearly indicated that toxic effects of anti-cancer therapy are long-lasting and could be detected as specific features of serum proteome many months after the treatment. Importantly, this finding indicates the possible applicability of MS-based analyses of serum proteome profiles in retrospective bio-dosimetry of radiation exposure. On the other hand, one should note that serum proteome components involved in a dose/volume-related “early” radiation response of the organism did not show significant association with long-term efficacy of the treatment (i.e. tumor eradication vs. re-growth) when a group of patients with similar prognosis treated with unified radiotherapy protocol was analyzed.

Model presented in current study is extremely complex and could be affected by many different processes ongoing in the patient’s organism. However, we assumed that accumulation and subsequent healing of radiation-induced damage (acute reactions, mucositis, hematological reactions, etc.) would have the major influence on general therapy-related changes observed at the level of serum proteome. In fact, we found an association of serum proteome features with the intensity of radiation-induced acute mucosal reaction, which was in accordance with the apparent correlation between the observed radiation toxicity and the doses of radiation delivered to normal tissues. We found the strongest correlation between the mucosal reaction and volume irradiated at 30-40 Gy (i.e., at 0.7-1.0 Gy dose fractions). Of note, changes of serum proteome profiles observed two weeks after the start of RT, which putatively accompanied escalation of mucosal reaction, also showed the most frequent correlations with volume of tissue irradiated at similar “intermediate” dose fractions (0.7-1.0 Gy). Several peptide ions registered in the low-molecular-weight fraction of serum proteome that changed their abundance in samples from irradiated patients were hypothetically annotated in the proteomic knowledge base EPO-KB [19] as fragments of proteins related to the inflammation, immunity and defense. Among such proteins, there were acute phase proteins (fibrinogen, haptoglobin, hepcidin, SAA), complement factors (CO3, CO4A), protease inhibitors (AACT, ANT3, CYTC, ITIH4), cytokines (CCL13, CXCL7, OSTP, PLF4, S10AC) and antibacterial defensins (DEF1, DEF3, DEFB1) (see Additional file 1: Table S1 and Table S7 for details). These processes are potentially involved in response to radiation, and several proteins related to acute phase and inflammation were previously reported in serum samples collected after radiotherapy of cancer patients [11]. Hence, we postulate that radiation-induced features of the serum proteome, detected in post-treatment samples, correspond to the direct toxic effects of radiation and/or healing of the acute reaction.

Due to dosimetrical superiority of IMRT plans this treatment modality constantly replaces the more “traditional” radiotherapy techniques. Here, we observed that irradiation of large volumes of normal tissue with doses of radiation considered as “therapeutically irrelevant”, which is typical for IMRT, may have some biological effects. Their potential importance for output of the treatment is not as obvious, because exposure of increased volume of normal tissues could hypothetically be either detrimental (e.g. inflammation reactions related to radiation toxicity) or beneficial (e.g. radiation-induced stimulation of immune reactions). Hence, their clinical implications need to be carefully considered when complete data of long-term effects concerning the IMRT implemented in large cohorts of patients are available for analyses.

## Conclusions

This is the first time that the effects of low and intermediate doses of radiation delivered to a large fraction of normal tissue have been documented at the level of the serum proteome. They apparently reflects the general response of patient’s organism to radiation, indicating potential biological relevance of therapeutically low doses during IMRT.

## Competing interests

The authors declare that they have no competing interests.

## Authors’ contributions

PW – designed the project, designed and interpreted experiments, prepared final manuscript. MP – performed experiments, interpreted results, JP – performed mathematical modeling and statistical analyses, TR - collected and interpreted clinical data, KJ – performed MS analyses, MKH – performed MS analyses, AGK – performed MS analyses, AW - collected and interpreted clinical data, RT - interpreted clinical part of the study, KS – designed and interpreted clinical part of the study. All authors read and approved the final manuscript.

## Supplementary Material

Additional file 1 Specific description of registered serum components. Click here for file
